# Combining *ε*-Near-Zero Behavior and Stopped Light Energy Bands for Ultra-Low Reflection and Reduced Dispersion of Slow Light

**DOI:** 10.1038/s41598-017-08342-x

**Published:** 2017-08-18

**Authors:** Frank Bello, A. Freddie Page, Andreas Pusch, Joachim M. Hamm, John F. Donegan, Ortwin Hess

**Affiliations:** 1 School of Physics and the Centre for Research on Adaptive Nanostructures and Nanodevices (CRANN), Trinity College Dublin, Dublin 2, Ireland; 2 Advanced Materials and Bioengineering Research (AMBER), Trinity College Dublin, Dublin 2, Ireland; 3 Blackett Laboratory, Department of Physics, Imperial College London, London, SW7 2AZ UK

## Abstract

We investigate media which exhibits epsilon-near-zero (ENZ) behavior while simultaneously sustaining stopped light energy bands which contain multiple points of zero group velocity (ZGV). This allows the merging of state-of-the-art phenomena that was hitherto attainable in media that demonstrated these traits separately. Specifically, we demonstrate the existence of Ferrell-Berreman (FB) modes within frequency bands bounded by points of ZGV with the goal to improve the coupling efficiency and localization of light in the media. The FB mode is formed within a double layer, thin-film stack where at subwavelength thicknesses the structure exhibits a very low reflection due to ENZ behavior. In addition, the structure is engineered to promote a flattened frequency dispersion with a negative permittivity able to induce multiple points of ZGV. For proof-of-concept, we propose an oxide-semiconductor-oxide-insulator stack and discuss the useful optical properties that arise from combining both phenomena. A transfer matrix (TM) treatment is used to derive the reflectivity profile and dispersion curves. Results show the ability to reduce reflection below 0.05% in accordance with recent experimental data while simultaneously exciting a polariton mode exhibiting both reduced group velocity and group velocity dispersion (GVD).

## Introduction

Low dispersion metamaterials which slow or stop light produce desirable properties in devices used for communications technologies^1, 2^. For example, various structures supporting more than one mode with ZGV have recently been proposed to self-trap light without the need for traditional feedback methods, specifically Bragg reflectors or carefully constructed photonic and plasmonic waveguides^3, 4^. Furthermore, metamaterials which are highly absorptive are beneficial in order to increase directional transmission while reducing any detrimental effects on the efficiency of an integrated device due to unwanted reflections^5^. We aim to enhance these features, i.e. to slow light, to lower dispersion and to reduce reflection, by taking advantage of asymmetric metamaterials that can support a frequency band bounded by ZGV points, i.e. stopped light bands, while simultaneously experiencing an ENZ material response.

ENZ materials are materials which can sustain permittivities (*ε*) well under the permittivity of free space and frequently below 0.1. This property, in combination with subwavelength film thicknesses, can be used to impedance match a superstrate with a metamaterial, stacked substrate by manipulating the phase shift of a wave as it travels through the stack^6^. The reduced reflection leads to an enhancement of the electric field in the structure which can in turn yield strong harmonic generation, soliton formation, as well as perfect absorption^7–11^. Within the past year alone, ENZ media have been produced using a variety of metamaterial structures ranging from multilayered metal-insulator films^12^, silicon pillar arrays^13^, and even a nonspecific family of soliton-like materials^14^. The latter is the first to our knowledge to report the coexistence of ENZ behavior for a mode which experiences ZGV. However, full exploitation of these optical effects have yet to be realized or fully understood in regards to their stability within integrated structures, in particular in anisotropic media where different components of the wavevector may experience a significantly altered material response. Herein, we focus on multilayered thin films for which an ENZ material response enhances the absorption of leaky bulk polaritons, known as Ferrell-Berreman modes, which lie within the light cone and may be radiatively excited^15–18^. These modes should not be confused with purely longitudinal bulk or volume plasmons which are not radiatively excited^18^. FB resonances have demonstrated an adaptable dispersion curve within multiple- or single-film materials^17^ and produce absorption within the visible to infrared wavelength range depending on layer thicknesses^19–21^.

Although it has recently been proven that a single point of ZGV is guaranteed in ENZ plasmonic metamaterials, there may be drawbacks for obtaining slow light near these ZGV points. Beyond being highly dispersive they are known to occur as the wavevector (*k*) asymptotically goes to zero, i.e. *ε* = 0, which yields a nonradiatively, very lossy mode^22–24^. Moreover, extremely small wavevectors are a limiting factor in regards to creating a device which can support guided modes of slow light and excite a material at its bandgap energy^25^. Therefore, we examine asymmetric structures which can be used to manipulate the energy bands of FB modes such that they are bounded by two ZGV points, i.e. stopped light bands, and merge the distinctive behavior of these bands with that of ENZ media. For instance, ZGV points within stopped light bands do not necessarily correspond to small wavevectors offering a number of subsequent benefits. In thin layer films, they resulted in the demonstration of cavity-free trapping of plasmon modes between ZGV points that provide feedback over a frequency range in which propagation of light is slow, thus mimicking the response of a cavity without the physical limitations of fabricating one^26^. Importantly, in contrast to a single ZGV point, stopped light bands are guaranteed to contain at least one point of zero frequency dispersion while in general multiple ZGV points promote a flattened dispersion as discussed below^27^. Indeed, a relatively flattened dispersion with multiple ZGV points was previously designed by combining bulk ITO and thin film Si layers^28^. It is also notable that at near-infrared wavelengths ITO not only has the required negative permittivity for producing slow light but at subwavelength thicknesses has been demonstrated to induce ENZ behavior with reduced reflection in multilayered stacks^7^. Therefore, we propose an asymmetric structure composed of nanoscale ITO and semiconductor material layers to efficiently absorb light whose frequency/momentum fall in a stopped light band^1, 29^.

## Results

### Reflection and absorption profiles

**Figure 1:**
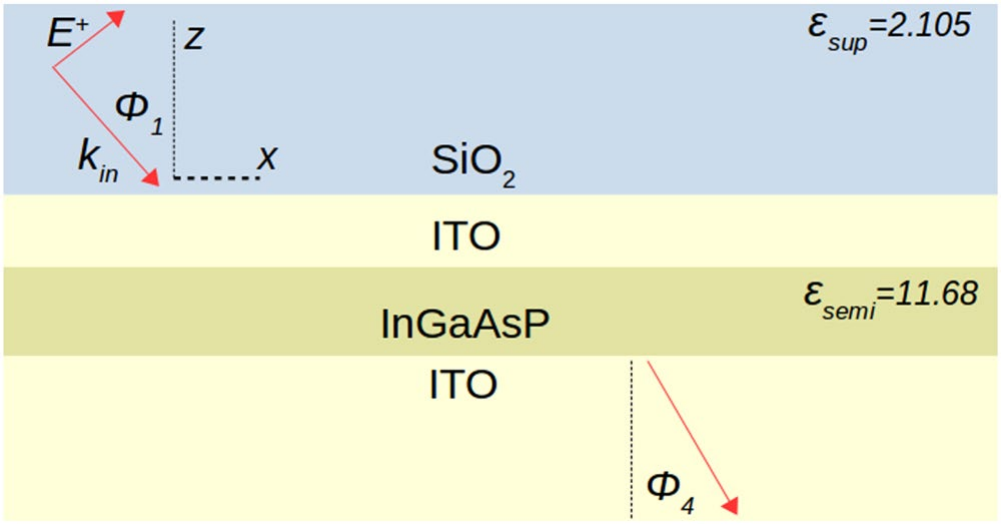
Schematic of the asymmetric metamaterial stack studied which is composed of Silicon Dioxide, Indium Tin Oxide, and Indium Gallium Arsenide Phosphide. Optimized thicknesses for the upper ITO and InGaAsP layers are 8 nm and 20.8 nm respectively. The incoming field, which is described later in the Methods section, is TM-polarized with an incident angle *φ*_1_.

A schematic of the multilayer structure investigated is shown in Fig. 1. Layers of Indium Gallium Arsenide Phosphide (InGaAsP) and Indium Tin Oxide (ITO) are deposited on a substrate of ITO and excited via a superstrate of Silicon Dioxide (SiO_2_). The thicknesses are optimized in order to excite an FB mode within a stopped light band that is robust to a few nanometers of surface roughness, overlaps with the bandgap energy of InGaAsP, and minimizes reflection as much as possible. We focus on plane wave excitation through the superstrate which is suitable if one is using SiO_2_ prism-based coupling via the Kretschmann configuration for experiments^30^.

**Figure 2:**
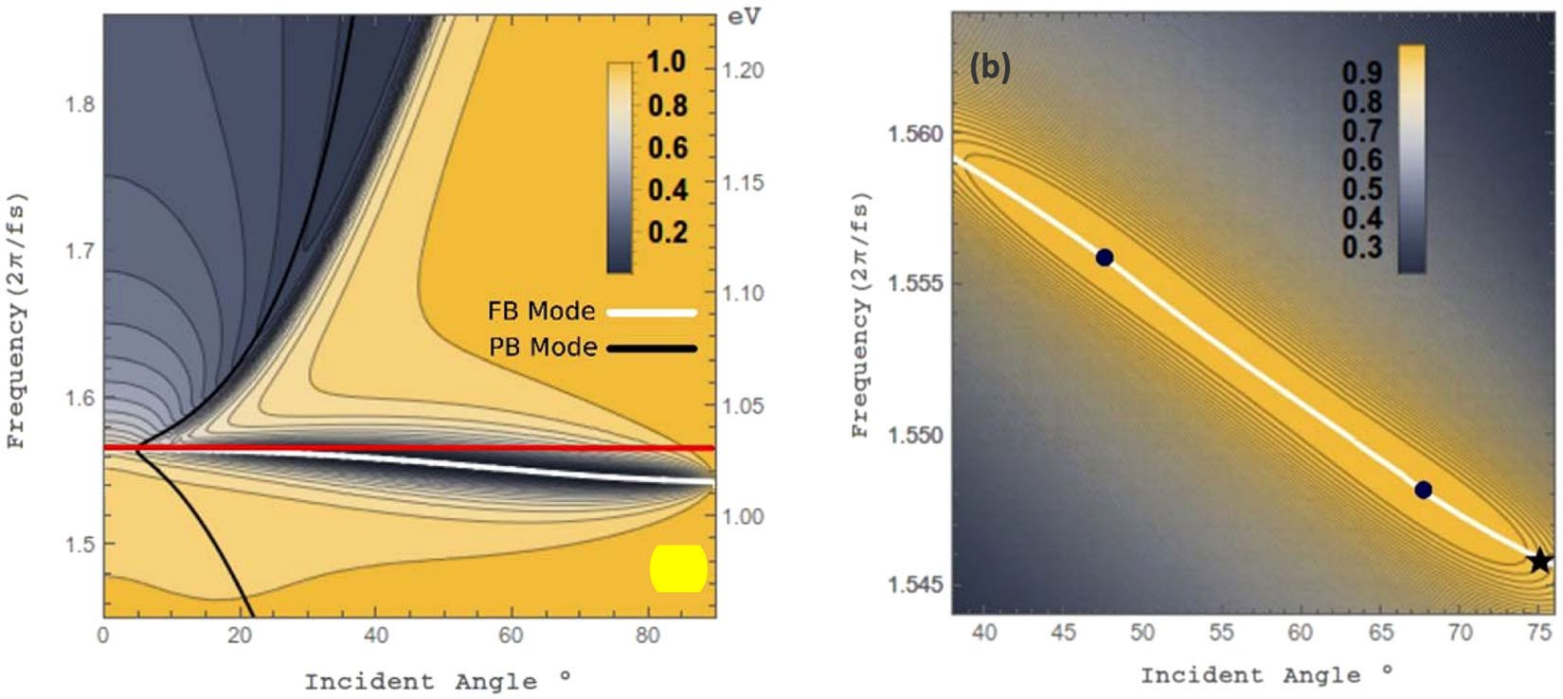
(**a**) Reflectivity profile as a function of incident angle (within SiO_2_) and input frequency. The dispersion of a bulk plasmon polariton mode (FB, white solid line) overlapping dip in reflectivity is predicted to occur due to ENZ behavior circa a frequency of 1.565 rad/fs (gray horizontal line, red online). Expected plasmon resonances for ITO thin films appear in the upper left of the profile andresonance excitation of a pseudo-Brewster (PB, black curve) mode is also superimposed. (**b**) The absorbance for the structure with values reaching over 98% within the innermost contour. The FB mode covers frequencies corresponding to a wavelength range of roughly 1207–1219 nm. Points of perfect antireflection (PAR) points and a point of zero GVD (black star) are highlighted.

Reflectivity results in Fig. 2(a) show a broad dip in reflectivity in the upper left corner of the profile. This plasmon resonance is anticipated in the near-infrared regime for ITO films utilizing a Drude-model treatment and it shifts towards values of the bulk plasmon frequency as ITO thicknesses are increased^31^. For reference, we have also superimposed the curve corresponding to the pseudo-Brewster (PB) angle (black curve) which is analogous to the Brewster angle however when calculating the PB angle, one considers the absorption of light incident on a lossy media with imaginary dielectric constants^32^. The bending-back nature of the reflectivity contours to the left of the profile is caused by the summing of reflectivity values from the InGaAsP-ITO and SiO_2_-ITO interfaces. Around 1.545–1.565 rad/fs, (≈1.02 eV) the narrow dip in reflection is attributed to a near-zero material response with the real and imaginary parts of the permittivity both nearing zero for ITO using a lossy Drude model treatment as we have for *ε*_*ITO*_. The absorbed mode is attributed to a radiative bulk plasmon polariton (white curve), also referred to as a Ferrell-Berreman mode, that exists to the left of the light line and disappears as film thicknesses are increased^17^. Upon examination, an uninterrupted contour for reflectivity reaches below 2.0% ranging roughly 39–74 degrees. In Fig. 2(b), the absorbance (*α*) is also shown but over a narrower frequency and incident angle range. The absorptance of is given by the emissivity in thermal equilibrium, $\alpha =1-{R}_{k,\Phi }-{T}_{k,\Phi }$, where *R* (*T*) refer to the spectral and directional reflectivity (transmission) powers^33^. For both PAR modes it is calculated to be >99.55%, with the peak intensity of absorbed power localized within the ITO thin film for the ENZ frequency (see supplementary material for details)^34, 35^.

**Figure 3:**
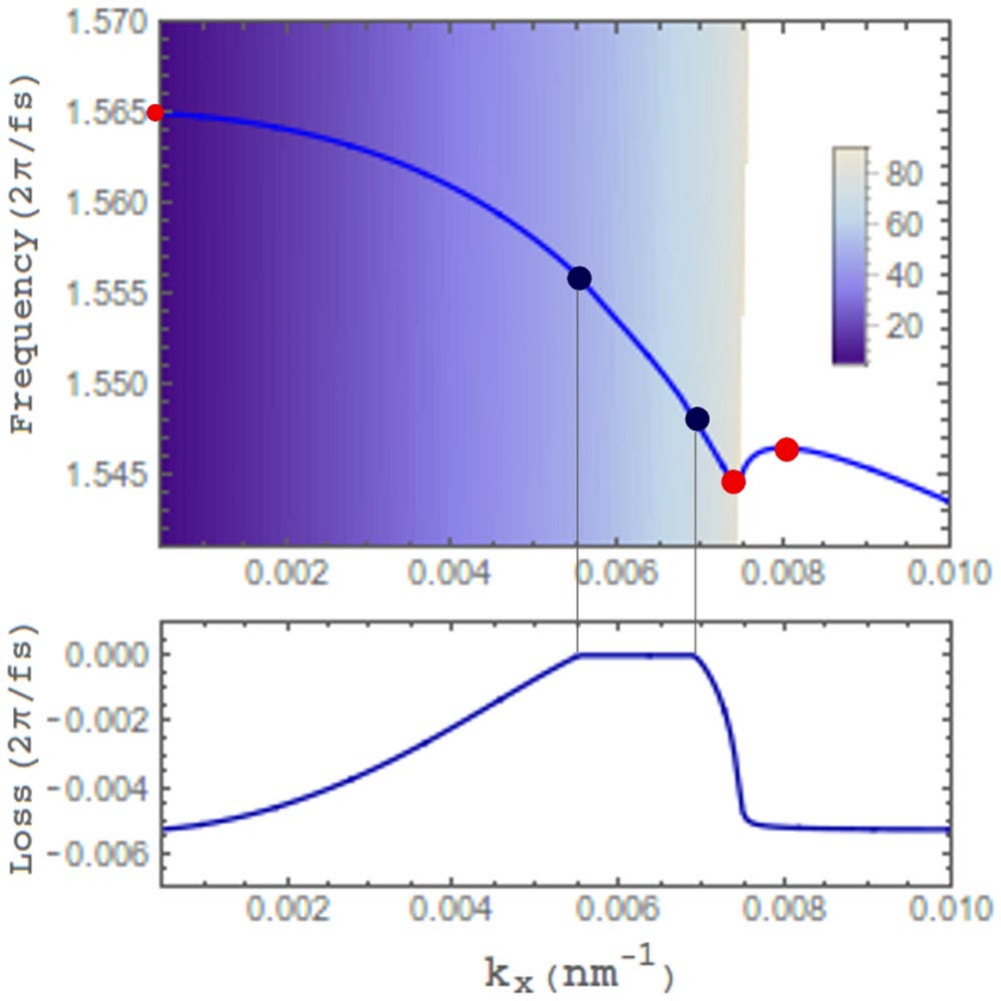
Dispersion curve of the FB mode with the shaded area corresponding to angles in the light cone (legend) and contains the part of the curve shown in the reflectivity profile of Fig. 1. Black dots (blue online) mark PAR points at k_x_ = 5.52 × 10^−3^ nm^−1^ and k_x_ = 6.9 × 10^−3^ nm^−1^. The three gray dots (red online) represent ZGV points. One lying at k_x_ = 0 at 1.565 rad/fs. The loss in the lower part of the diagram corresponds to the imaginary part of the complex frequency.

Reflection is expected to be further reduced for an incident wave if one can phase match it with a FB mode. Using a complex frequency analysis, the FB dispersion curve of Fig. 2 is derived and plotted in Fig. 3 ^28^. Indeed, two minima are found on the reflectivity profile which corresponds to purely real frequencies on the dispersion curve and therefore an incoming wave can be perfectly phase matched and absorbed^30^. The reflection minima or points of perfect antireflection (PAR) are highlighted on the dispersion curve (black dots, blue online) and defined as having reflectivity values below 0.05%. They are obtained at energies of 1.556 and 1.548 rad/fs corresponding to incident angles of 47.2° and 67.9° respectively. These incident frequencies for PAR agree well with near-infrared measurements of perfect absorption in ITO films and may be shifted relative to each other by varying ITO thickness^7, 10^. The radiative loss of these modes (e^−*ω*t^ dependent) is due to a non-zero imaginary part of the frequency and shown as negative values in Fig. 3. It is dominantly Ohmic in particular when phase matching an incoming wave (ω″ = 0). However, near points of PAR, low reflectivity is still expected considering the relatively low radiative losses^36, 37^ and therefore we see the elongated dip in reflectivity overlapping with the FB mode. As the FB mode transitions to a bound mode of the metamaterial, the loss returns to *γ*/2 (5.35 × 10^−3^ rad/fs) for in-plane modes outside the light cone. Near this point, just before the light line of SiO_2_, a change in curvature and ZGV point occur, marked by a red dot (gray online), while two other ZGV points which form stopped light bands are found to occur shortly beyond the light line and as k_x_ goes to zero. For practical applications dissipative loss is a crucial issue to be addressed. To tackle this issue the layer of semiconducting InGaAsP is not only able to promote a flattened dispersion curve in combination with ITO^28^ but it allows gain to be introduced into the metamaterial stack^3^. Supplementary material is provided demonstrating how the loss in the FB mode can be affected when gain is introduced within the InGaAsP layer. It should be noted that the input frequency for PAR agrees well with bandgap values for InGaAsP near 1.02 eV^38^.

The figure of merit (*f)* for stopped light devices balances the range of *k*-vectors with the flatness of the band by calculating the average band velocity. The structure presented yields *f* = |Δ*k*_*x*_/Δ*ω*| ≈ 0.4; previously established to be a good candidate for stopped light lasing where structures on the same order (*f* ≈ 1) have been shown to lase with significantly reduced threshold pumping^2, 3, 27^.

### Results on group velocity and GVD

**Figure 4:**
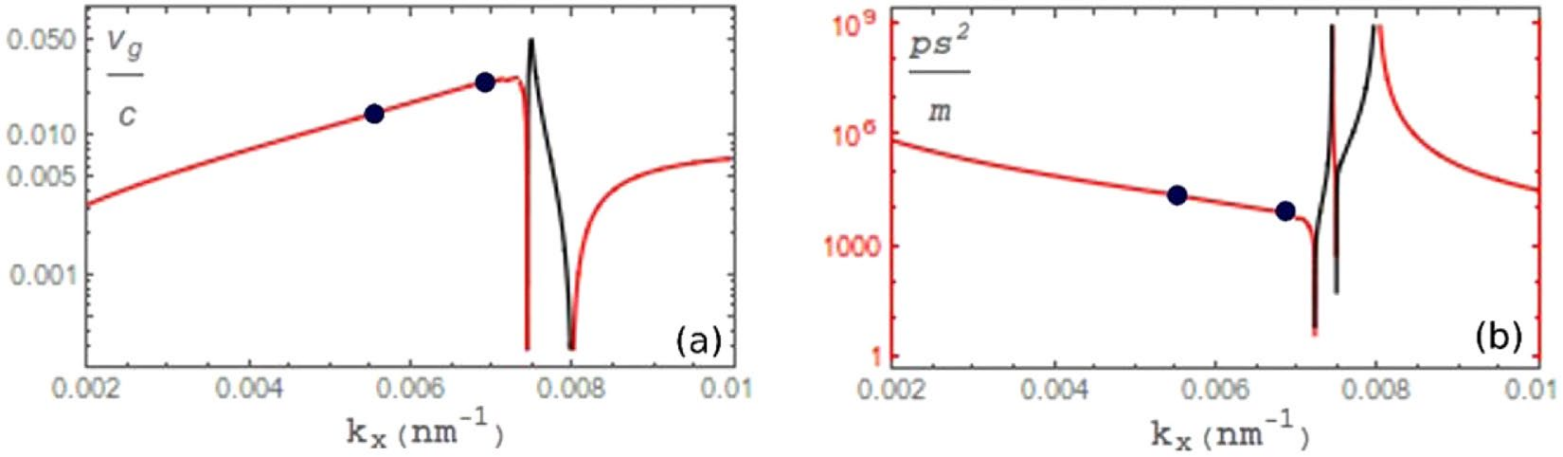
(**a**) Absolute value of the group velocity of the FB mode with negative values of the group velocity in gray (red online) and positive values in black. (**b**) Absolute value of the GVD is shown with transitions between normal (red online, gray) and anomalous dispersion (black). PAR points are highlighted.

The radiative FB mode prominently lies within a stopped light energy band, bounded ZGV points, which spans the entire frequency range inside the light cone. The group velocity, Re[${\nabla }_{k}\omega$], is plotted in Fig. 4(a) along with the group velocity dispersion (GVD), Re[${\nabla }_{\omega }^{2}k$], in Fig. 4(b). For small in-plane wavevectors the group velocity is reduced to the order of 10^−3^ *c* yielding propagation lengths (2v_g_/ω″) between 1–1.5 μm. When phase matching an incident plane wave (black dots), light will slow to roughly 0.02c which is comparable to other plasmonic devices designed for slow light operation^39, 40^. Here the GVD is also similar to previous HMM devices on the order of 10^4^ ps^2^/m; highly useful in dispersion compensation devices but now additionally combined with PAR^41, 42^. One can notice the large dispersion near individual ZGV points, however slow light around 0.02*c* and zero GVD exists just before the light line within the stopped light band (highlighted in Fig. 2(b)). At points of ZGV the dispersion switches from normal (red curve online) to anomalous (black) or vice versa. Unlike previous plasmonic ENZ materials that guarantee a ZGV point at k_x_ = 0, the two additional ZGV points near the light line are accompanied by a change in the sign of group velocity. Since the phase velocity (ω′/k_x_) of the FB mode is always positive, we therefore have group and phase velocities in opposing directions signifying a refractive index that is switching between positive and negative refraction. The wide range of tuning available for the refractive index, combined with reduced dispersion and low reflectivity, allows for a number of applications to be exploited such as nanoscale heat transfer, antireflective coatings, and subdiffraction focusing elements to name a few^43–45^. Furthermore, one may take advantage of enhanced harmonic generation, spatial chirping of non-periodic stacks, or the recently verified electrical tuning of modes in ITO layers in order to access areas of ZGV and zero or negative GVD to go along with excellent absorption^27, 46–49^.

## Discussion

To conclude, we have demonstrated the existence of FB modes within stopped light bands using multilayered thin-films. By combining ENZ behavior with a stopped-light energy band structure we are able to permeate slow light throughout the mode’s band structure which simultaneously yield zero GVD and ultra-low reflection. This unified behavior yielded by the asymmetric thin-film layers and subsequent novel results have yet to be produced elsewhere to the best of our knowledge. The asymmetric stack is able to produce slow and low dispersive light along with a much reduced reflectivity below 0.05% by taking advantage of the impedance matching behavior of an ENZ material and phase matching with a FB mode. Although the dissipative loss is not negligible, the embedded semiconducting material enables gain to be introduced in the structure whose stopped light band overlaps well with the bandgap energy of InGaAsP^26–28^. The subwavelength structure allows for integration within coupled photonic and plasmonic devices along with a number of available tuning mechanisms for the plasmon’s momentum previously exhibited in ITO films^7, 10, 47^. This is on top of the aforementioned benefits of ENZ media and stopped light bands with frequency tuning between platforms yielding a multi-purpose metamaterial.

## Methods

### Transfer matrix analysis

The field is represented by a TM-polarized plane wave, ${E}_{i}={E}_{i}^{+}{{\rm{e}}}^{{{\rm{ik}}}_{{z}_{i}}{{\rm{d}}}_{i}}+{E}_{i}^{-}{{\rm{e}}}^{-{{\rm{ik}}}_{{z}_{i}}{{\rm{d}}}_{i}}$, where the +(*−*) sign represents the forward (backward or reflected) propagating wave at the boundary interface of layers *i* and *i* + *1* with thicknesses *d*_*i*_, of 20.8 and 8 nm for InGaAsP and ITO layers, respectively. The complex wavevector normal to the incident plane is defined as ${k}_{zi}=\sqrt{{\varepsilon }_{i}{\omega }^{2}/{c}^{2}-{k}_{x}^{2}}$, while ${{\rm{k}}}_{{\rm{x}}}={k}_{in}\sqrt{{\varepsilon }_{1}}\,\sin \,{\phi }_{1}$ is taken to be real and conserved at each boundary. The permittivity of ITO is defined within the Drude free-electron model, ${\varepsilon }_{ITO}={\varepsilon }_{\infty }-{\omega }_{p}^{2}/({\omega }^{2}+i\omega \gamma )$, with designations for the plasma frequency (ω_*p*_ = 3.13 rad/fs), scattering rate (γ = 1.07 × 10^−2^ rad/fs)^3, 50^, and background permittivity (*ε*_∝_ = 4.0). It should be noted that various factors such as surface roughness as well as non-local responses due to quantum pressure and diffusion may considerably increase the scattering rate^51–54^. The effects on the dispersion curve from an increased scattering rate compared to the local limit we have used are addressed in the accompanying supplementary material. The reflectivity profile shown in Fig. 2 is extracted from the transfer matrix of the HMM stack which relates the reflected and transmitted fields at each surface boundary and is defined as,

1 $$(\begin{array}{c}{{\boldsymbol{E}}}_{{\boldsymbol{i}}}^{+}{{\boldsymbol{e}}}^{{\boldsymbol{i}}{{\boldsymbol{k}}}_{zi}{{\boldsymbol{d}}}_{{\boldsymbol{i}}}}\\ {{\boldsymbol{E}}}_{{\boldsymbol{i}}}^{-}{{\boldsymbol{e}}}^{-{\boldsymbol{i}}{{\boldsymbol{k}}}_{zi}{{\boldsymbol{d}}}_{{\boldsymbol{i}}}}\end{array})=(\begin{array}{cc}1/{{\boldsymbol{t}}}_{{\boldsymbol{i}},{\boldsymbol{i}}+1} & {{\boldsymbol{r}}}_{{\boldsymbol{i}},{\boldsymbol{i}}+1}/{{\boldsymbol{t}}}_{{\boldsymbol{i}},{\boldsymbol{i}}+1}\\ {{\boldsymbol{r}}}_{{\boldsymbol{i}},{\boldsymbol{i}}+1}/{{\boldsymbol{t}}}_{{\boldsymbol{i}},{\boldsymbol{i}}+1} & 1/{{\boldsymbol{t}}}_{{\boldsymbol{i}},{\boldsymbol{i}}+1}\end{array})(\begin{array}{c}{{\boldsymbol{E}}}_{{\boldsymbol{i}}+1}^{+}{{\boldsymbol{e}}}^{{\boldsymbol{i}}{{\boldsymbol{k}}}_{zi+1}{{\boldsymbol{d}}}_{{\boldsymbol{i}}}}\\ {{\boldsymbol{E}}}_{{\boldsymbol{i}}+1}^{-}{{\boldsymbol{e}}}^{-{\boldsymbol{i}}{{\boldsymbol{k}}}_{zi+1}{{\boldsymbol{d}}}_{{\boldsymbol{i}}}}\end{array})$$

The transfer matrix (*M*_*i*_,_*i*+1_) between successive layers is defined by a 2 × 2 matrix whose elements consist of the reflectivity (*r*) and transmission (*t*) coefficients at each interface.

2 $$\,{{\boldsymbol{t}}}_{{\boldsymbol{i}},{\boldsymbol{i}}+1}=\frac{2{\sqrt{{\boldsymbol{\varepsilon }}}}_{{\boldsymbol{i}}}\,{\cos }\,{{\boldsymbol{\Phi }}}_{{\boldsymbol{i}}}}{{\sqrt{{\boldsymbol{\varepsilon }}}}_{{\boldsymbol{i}}+1}\,{\cos }\,{{\boldsymbol{\Phi }}}_{{\boldsymbol{i}}}+{\sqrt{{\boldsymbol{\varepsilon }}}}_{{\boldsymbol{i}}}\,{\cos }\,{{\boldsymbol{\Phi }}}_{{\boldsymbol{i}}+1}}$$

3 $${{\boldsymbol{r}}}_{{\boldsymbol{i}},{\boldsymbol{i}}+1}=\frac{{\sqrt{{\boldsymbol{\varepsilon }}}}_{{\boldsymbol{i}}+1}\,{\cos }\,{{\boldsymbol{\Phi }}}_{{\boldsymbol{i}}}-{\sqrt{{\boldsymbol{\varepsilon }}}}_{{\boldsymbol{i}}}\,{\cos }\,{{\boldsymbol{\Phi }}}_{{\boldsymbol{i}}+1}}{{\sqrt{{\boldsymbol{\varepsilon }}}}_{{\boldsymbol{i}}+1}\,{\cos }\,{{\boldsymbol{\Phi }}}_{{\boldsymbol{i}}}+{\sqrt{{\boldsymbol{\varepsilon }}}}_{{\boldsymbol{i}}}\,{\cos }\,{{\boldsymbol{\Phi }}}_{{\boldsymbol{i}}+1}}$$

The transfer matrix of the entire structure considers reflection and transmission at each boundary interface and is defined as ${\rm{T}}=\prod _{{\rm{i}}}{{\rm{M}}}_{{\rm{i}},{\rm{i}}+1}$. We then subsequently compute the ratio of reflected and incident field amplitudes at the superstrate-ITO interface, $|{E}_{1}^{-}/{E}_{1}^{+}|$ ^9^.

To show the correspondence between the reflectivity and near-zero permittivity of the ITO film, we examine the conditions for PAR based on a Fresnel equation approach. For simplicity the under layers are approximated as perfectly conducting yielding the following two conditions for the input wavelength (*λ*) and incident angle which must be simultaneously met^7^,

4 $${\sqrt{{\boldsymbol{\varepsilon }}}}_{1}\,{\sin }\,{{\boldsymbol{\Phi }}}_{1}=\sqrt{\frac{{|{{\boldsymbol{\varepsilon }}}_{{\boldsymbol{ITO}}}|}^{2}}{{{\boldsymbol{\varepsilon }}}_{{\boldsymbol{ITO}}}^{^{\prime} }}}$$

5 $$\frac{2{\boldsymbol{\pi }}{{\boldsymbol{d}}}_{{\boldsymbol{ITO}}}}{{\boldsymbol{\lambda }}}=\frac{{{\boldsymbol{\varepsilon }}}_{{\boldsymbol{ITO}}}^{^{\prime} }\,{\cos }\,{{\boldsymbol{\Phi }}}_{1}}{{{\boldsymbol{\varepsilon }}}_{{\boldsymbol{ITO}}}^{^{\prime\prime} }{\sqrt{{\boldsymbol{\varepsilon }}}}_{1}}$$

where ${\varepsilon }_{ITO}^{^{\prime} }$ (${\varepsilon }_{ITO}^{^{\prime\prime} }$) is the real (imaginary) part of the ITO permittivity. From eqn. (5) one can notice that the equality is satisfied if the thickness of the thin film ITO layer and the real component of its permittivity both approach zero. Consequently eqn. (4) tells us that the imaginary part of the ITO permittivity must also approach zero in order to prevent the radical from diverging.

**Figure 5:**
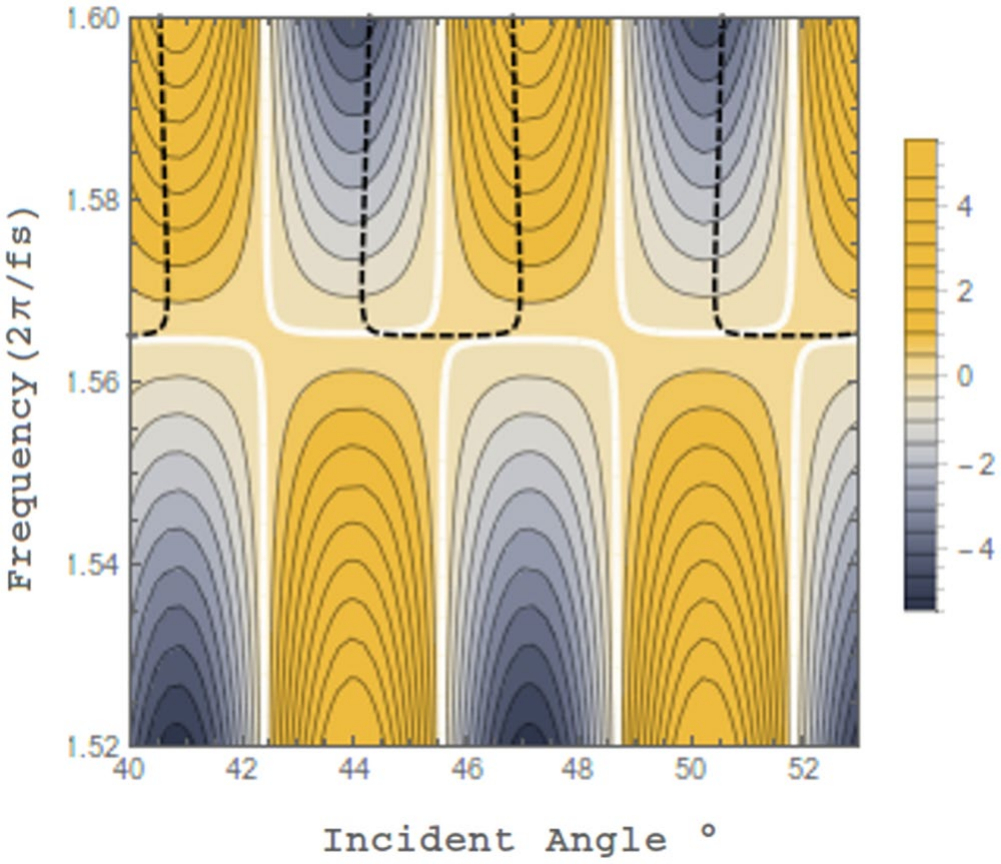
Plotted is the deviation from the PAR condition represented by the equality in eqn. (5). The black (dashed) and white lines denote values for which the frequency and incident angle satisfy the PAR conditions of eqns (4) and (5) respectively. Crossing points of the white and black dashed lines which satisfy the conditions for PAR agree well with the narrow dip in reflectivity of Fig. 2 between 1.55–1.57 rad/fs.

Figure 5 shows that the conditions for PAR are met for a number of incident angles. Here we have plotted the deviation from the condition represented by eqn. (5) with the white line signifying the values of frequency and incident angle which match the condition exactly. Overlapped on this plot are the values for which the condition represented by eqn. (4) is also satisfied (black dashed line). The black and white lines periodically intersect at points where the PAR conditions are fulfilled. Between these points, circa a frequency of 1.565 rad/fs, the PAR condition is only slightly deviated from and therefore reflection is still anticipated to be extremely low, agreeing well with the reflectivity profile. Using an ITO thickness of 8 nm we find the condition for PAR is predicted to occur for a wavelength of *λ* *≈* 1204 nm (≈1.565 rad/fs) for which ${\varepsilon }_{ITO}^{^{\prime} }$ = 0.002 and ${\varepsilon }_{ITO}^{^{\prime\prime} }$ = 0.027; delineated by a gray horizontal line in Fig. 2(a) (red online). This agrees precisely with the TM results at normal incidence. It should be noted that as one moves above and below this energy we begin to strongly deviate from the PAR conditions of eqns (4) and (5) where we also expect a large amount of reflected power from the bulk ITO substrate. In addition, it is the thin films which dominate the structure of the dispersion curve and reflectivity profile (see supplementary materials for an analysis of the effect of ITO thickness on absorption). The super and substrate materials are optimized for radiative coupling of the FB mode and to minimize transmission. The dispersion curve of Fig. 3 is derived by taking the frequency with the TM analysis to be complex and assuming the plasmon decays away as one moves away from the stack in the super and substrate^26, 28^.

## Electronic supplementary material

Supplementary Information
