# Supplementary material for: Combining ε-Near-Zero Behavior and Stopped Light Energy Bands for Ultra-Low Reflection and Reduced Dispersion of Slow Light
Source: Sci Rep. 2017 Aug 18;7:8702. doi: 10.1038/s41598-017-08342-x (PMC5562864; doi:10.1038/s41598-017-08342-x)
Supplement: Supplementary file 1 — Supplementary Information [file 41598_2017_8342_MOESM1_ESM.pdf]

Supplementary Material For

Combining ε-Near-Zero Behavior and Stopped Light Energy Bands for Ultra-Low Reflection and Reduced Dispersion of Slow Light

Frank Bello,[1,2*] A. Freddie Page,[3] Andreas Pusch,[3] Joachim M. Hamm,[3] John F. Donegan[1,2], and Ortwin Hess[3]

[1]*School of Physics and the Centre for Research on Adaptive Nanostructures and Nanodevices (CRANN), Trinity College Dublin, Dublin 2, Ireland*

[2]*Advanced Materials and Bioengineering Research (AMBER), Trinity College Dublin, Dublin 2, Ireland*

[3]*Blackett Laboratory, Department of Physics, Imperial College London, London SW7 2AZ, U.K.*

**Corresponding author: fbello@tcd.ie*

Effect of gain within InGaAsP on Ferrell-Berreman mode

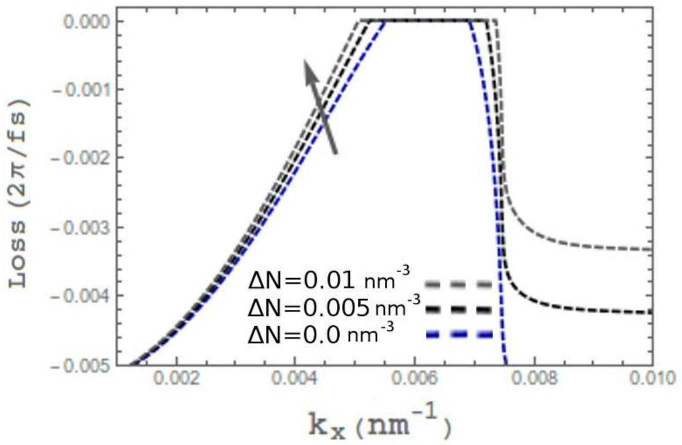

Figure S1. The change in the imaginary part of the complex frequency (ω'') of the Ferrell-Berreman (FB) mode is shown for increasing gain densities (ΔN) within the InGaAsP layer (following the direction of the arrow).

To consider gain within the InGaAsP layer a Drude-Lorentz model was used in order to account for the pumping between two levels with energy difference $\hbar\omega_e$ with permittivity defined as $\varepsilon_{InGaAsP} = \varepsilon_{bg} - \omega_{pe}^2/(\omega^2 + i\omega\gamma_e - \omega_e^2)$ and $\omega_{pe}^2 = \Delta N\sqrt{\varepsilon_{bg}}\gamma_e\sigma_e c$ [3,26]. Values for thin film InGaAsP are defined as:

background permittivity ε_{bg} = 11.68, emission frequency $\sigma_e = 778$ ps^{-1}, emission cross section 0.212 nm^2, and emission width $\gamma_e = 31.7$ ps^{-1} [3,27]. Pumping is considered by adjusting the gain density (ΔN) in the InGaAsP structure. In Fig. S1 the radiative loss (ω'') of the Ferrell-Berreman mode is shown to decrease as gain in the InGaAsP layer is increased while the range of k_x values for which the mode is phase matched with incident light is also increased. For similar figures of merits (*f*=0.4) reported in the text, sufficient pumping in ITO-InGaAsP structures has been reported to even undamp the plasmonic mode and induce lasing around the gain region [27,28]. For an in depth analysis of lasing within stopped light bands, the time evolution dynamics due to gain are presented in [3].

Effect of increased scattering rate in ITO on dispersion curve

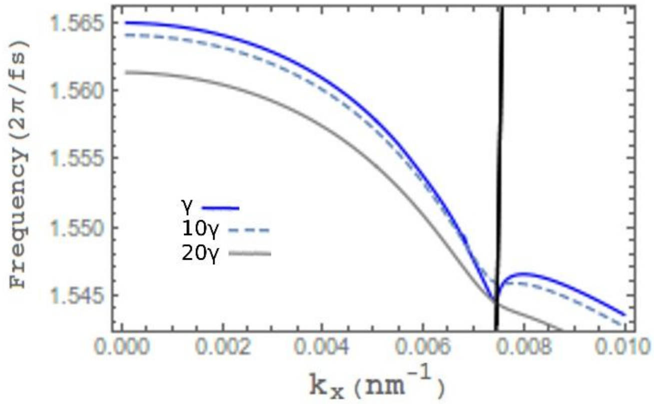

Figure S2. The adjustment to the dispersion curve (ω') as a function of increasing scattering rate in ITO.

A number of factors could contribute to increasing the loss within ITO films, namely scattering effects due to surface roughness or ballistic transport as well as non-local responses due to diffusion and quantum pressure [51-54]. Values for the non-local response in particular may adjust the loss by many times if the ITO thickness is on the order of a few nanometers [S1]. The increase in scattering is also dependent on the concentration of Indium and Tin which has a strong effect on Fermi velocity and mean free path [S2]. When adjusting the loss by a full order of magnitude, values circa PAR points for GVD (10^4 ps^2/m) and group velocity ($10^{-2}c$) remain on the same order with a change in resonance frequency less than 0.1%. As changes to the scattering rate go above an order of magnitude (20γ, gray curve), the point of zero-group velocity circa the light line is lost though values for the group velocity are still $10^{-3}c$ at this point. For scattering rates well over an order of magnitude larger than the local-response value we have used in the text, consideration should be given to the optimal layer thicknesses needed to achieve the PAR condition as defined within Eqns. 4 and 5. It should be noted that as the loss increases the thickness requirement for PAR also increases in which case the contribution from non-local responses of the thin film should be alleviated [7].

Absorption profile for varying ITO thin-film thicknesses

Given fabrication constraints and variations in surface roughness typically on the order of a few nanometers or less in ITO films [53], we have demonstrated changes to the absorption profile for increasing ITO thicknesses of +5 (13 nm) and +10 nm (18 nm) in Fig. S3. Perfect absorption due to ENZ behavior has been experimentally demonstrated in ITO films up to ≈100 nm thick on glass

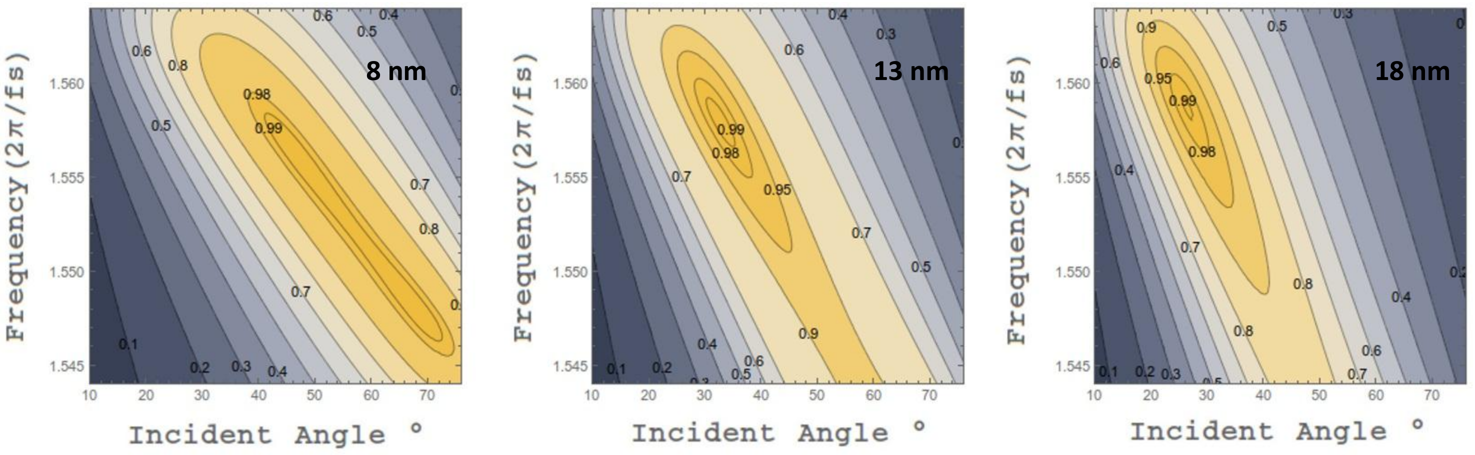

Figure S3. Absorption contour profiles for varying ITO film thicknesses of 8, 13 and 18 nm (left to right).

while third harmonic enhancement has been measured in ITO films ≈ 40 nm thick on glass [7,10]. As described in the Methods section, a change in ITO film thickness predicts that an adjustment to the incident angle needs to be made to achieve PAR if all other parameters are kept the same. The dimensions we have chosen were optimized to achieve ultra-low reflection over a wide range of angles in addition to obtaining slow light and reduced dispersion within the band structure.

Electric field distribution for slow light at the ENZ frequency

In order to demonstrate the field localization within the structure, we use Comsol software to simulate the 3D Helmholtz equation using a finite-element method along with the parameters given in the text for the permittivities of each material [S3]. No gain is included in the InGaAsP layer. Using a real TM plane wave for the incident wave travelling through SiO_2 as described in the schematic of Fig. 1 of the text, we plot the normalized electric field at an incident angle corresponding to a point of zero group velocity (86 degrees) at the ENZ frequency of 1.51 rad/fs. This frequency showing ENZ absorption corresponds to a shift of about 2% from the projected ENZ frequency at 1.545 rad/fs for this incident angle from the transfer matrix analysis. At the ENZ frequency the majority of power is predicted to be localized in the thin film. The wave vector k_x experiences a slow group velocity such that the power flow in this direction is many times greater than the normal component. This has been attributed to the quasi-trapping of slow light in regions of reduced group velocity [34,35].

Red arrows plot the direction of power flow (Poynting vector) with the size of the arrow being proportional to its magnitude. For stopped light the energy flow should cancel leading to a closed-loop vortex as we see here and originally predicted in Ref. [1,3]. This general behavior occurs throughout the stopped light band although the vortex tends to break down away from the ZGV point. The field distribution for completely stopped light has been demonstrated to remain spatially constant with the intensity decaying away in time [28].

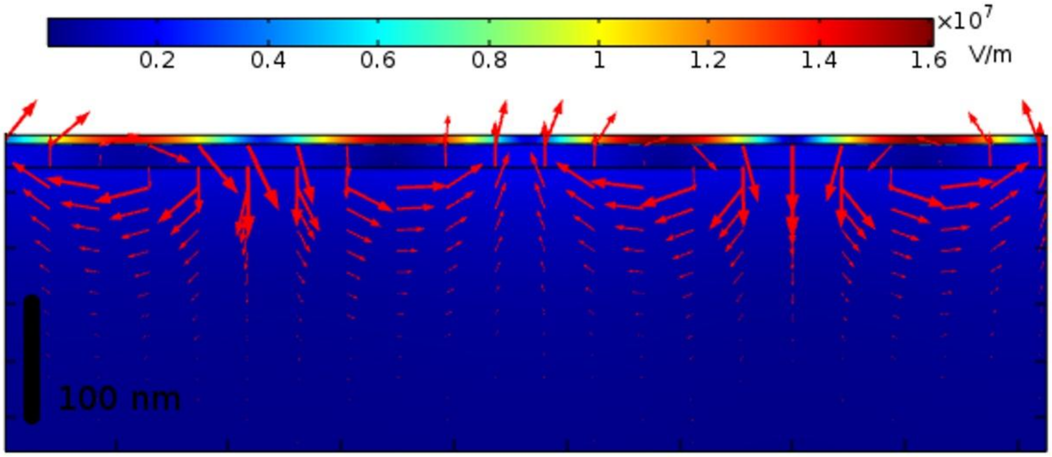

Figure S4. The normalized electric field distribution along with the direction of power flow (Poynting vector, red arrows) is shown throughout the metamaterial where an incident TM plane wave travelling in SiO_2 was used with an input power of 10 W. Data was taken for an incident angle of 86 degrees and input frequency 1.51 rad/fs. The top layer is the 8 nm ITO thin film. The substrate thickness was chosen such that scattering from the lower boundary has a negligible effect on the field distribution within the thin films. The edges are taken to be perfect electric conductors (PECs).

Supplementary References

[S1] Y. Yang, B. Zhen, C. W. Hsu, O. D. Miller, J. D. Joannopoulos, and M. Soljačić, “Optically thin metallic films for high-radiative-efficiency plasmonics” Nano Lett. **16**, 4110-4117 (2016).

[S2] S. H. Brewer and S. Franzen, “Calculation of the electronic and optical properties of Indium Tin Oxide by density functional theory”, J. Chem. Phys. **300**, 285–293 (2004).

[S3] COMSOL Multiphysics™ v. 5.2a www.comsol.com. COMSOL AB, Stockholm, Sweden.
